# Suppression of TGFβ-Induced Interleukin-6 Secretion by Sinulariolide from Soft Corals through Attenuation of the p38–NF-kB Pathway in Carcinoma Cells

**DOI:** 10.3390/ijms241411656

**Published:** 2023-07-19

**Authors:** Jenq-Lin Yang, Weng-Ling Lin, Shun-Ban Tai, Yi-Siang Ciou, Chih-Ling Chung, Jih-Jung Chen, Pei-Feng Liu, Ming-Wei Lin, Chun-Lin Chen

**Affiliations:** 1 Institute for Translational Research in Biomedicine, Kaohsiung Chang Gung Memorial Hospital, Kaohsiung 83301, Taiwan; 2 Department of Pathology, Kaohsiung Armed Forces General Hospital, Kaohsiung 80284, Taiwan; 3 Department of Biological Sciences, National Sun Yat-Sen University, Kaohsiung 80424, Taiwan; 4 Division of Rheumatology, Immunology and Allergy, Department of Internal Medicine, Zuoying Branch of Kaohsiung Armed Forces General Hospital, Kaohsiung 81342, Taiwan; 5 Department of Pharmacy, School of Pharmaceutical Sciences, National Yang Ming Chiao Tung University, Taipei 112304, Taiwan; 6 Department of Medical Research, China Medical University Hospital, China Medical University, Taichung 404332, Taiwan; 7 Department of Biomedical Science and Environmental Biology, Kaohsiung Medical University, Kaohsiung 80756, Taiwan; 8 Department of Medical Research, E-Da Hospital/E-Da Cancer Hospital, Kaohsiung 82445, Taiwan; 9 Department of Biotechnology, Kaohsiung Medical University, Kaohsiung 80708, Taiwan; 10 Graduate Institute of Natural Products, College of Pharmacy, Kaohsiung Medical University, Kaohsiung 80756, Taiwan

**Keywords:** sinulariolide, TGF-β, interleukin-6

## Abstract

Sinulariolide (SC-1) is a natural product extracted from the cultured-type soft coral *Sinularia flexibilis* and possesses anti-inflammation, anti-proliferative, and anti-migratory in several types of cancer cells. However, the molecular pathway behind its effects on inflammation remains poorly understood. Since inflammatory cytokines such as TGFβ, TNFα, IL-1, IL-6, and IL-8 activate transcription factors such as Smads, NF-κB, STAT3, Snail, Twist, and Zeb that drive the epithelial-to-mesenchymal transition (EMT), in this study, we focus on the investigation in effects of SC-1 on TGFβ-induced interleukin-6 (IL-6) releases in an in vitro cell culture model. We showed that both intracellular IL-6 expression and secretion were stimulated by TGFβ and associated with strong upregulation of IL-6 mRNA and increased transcription in A549 cells. SC-1 blocked TGFβ-induced secretion of IL-6 while showing no effect on the induction of fibronectin and plasminogen activator inhibitor-1 genes, indicating that SC-1 interferes with only a subset of TGFβ activities. In addition, SC-1 inhibits TGFβ-induced IL-6 by suppressing p38 MAPK signaling and subsequently inhibits NF-κB and its nuclear translocation without affecting the canonical Smad pathway and receptor turnover. Overall, these data suggest that p38 may involve in the inhibition of SC-1 in IL-6 release, thus illustrating an inhibitory effect for SC-1 in the suppression of inflammation, EMT phenotype, and tumorigenesis.

## 1. Introduction

Marine soft corals are plentiful of biologically active substances with bioactive functions, such as anti-inflammatory, anti-cancer, anti-fungal, antiviral, and cytotoxicity effects [1]. Sinulariolide (SC-1) is an active compound extracted from cultured soft coral *Sinularia flexibilis*. SC-1 has been shown to inhibit cell proliferation and induce apoptosis through the p38 MAPK pathway in bladder cancer [2]; it has also been found to inhibit the growth of melanoma, hepatoma, and lung adenocarcinoma cell lines through a mitochondria-related pathway and an ER-stress pathway [3,4,5,6]. Other studies have shown that SC-1 has weak to moderate cytotoxicity against various cancer cell lines and has significant anti-inflammatory effects by inhibiting the accumulation of pro-inflammatory inducible nitric oxide synthase (iNOS) and COX-2 proteins in lipopolysaccharide-simulated RAW264.7 macrophage cells [7,8]. SC-1 has also been shown to reduce the release of interleukin (IL)-6, IL-12, tumor necrosis factor-α, and nitric oxide from lipopolysaccharide (LPS)-activated dendritic cells, thereby reducing their ability to stimulate allogeneic T cells and inhibiting the LPS-induced nuclear factor B pathway [9].

A growing body of evidence indicates that cancer development is strongly influenced by both acute and chronic inflammation processes [10]. Recent research on inflammation has revealed links between inflammatory processes and neoplastic transformation, tumor progression, cancer cell dissemination, and recurrence [11]. Concentrations of the immunosuppressive cytokine TGFβ and the immunostimulatory cytokine IL-6 are increased in advanced stages of cancer and are strongly associated with poor prognosis in cancer patients. This initially counterintuitive concept actually seems to fit with experimental data, in which IL-6 and TGFβ orchestrate an EMT-permissive tumor microenvironment and tumor metastasis [12,13,14] and make cells more resistant to chemotherapy [15].

Although considered primarily anti-inflammatory, TGFβ contributes to the inflammatory environment of tumor mediators and cell types, promotes tissue remodeling, and directly and locally inhibits antigen-specific CD8+ T cell function [16]. Previous studies have demonstrated that the IL-6 gene expression is activated by tumor-producing TGFβ in many cell types, including human prostate cancer cells, fibroblasts, osteoblasts, and retinal pigmented epithelial cells [17,18,19,20,21,22,23,24]. Park et al. also showed that elevated IL-6 plays an important role in the oncogenic transformation of TGFβ in prostate tumorigenesis, in part by counteracting the growth-inhibitory effects of TGFβ [17].

TGFβ cytokines consist of three isoforms, TGFβ 1, 2, and 3, which are secreted and sequestered extracellularly as inactive homodimeric polypeptides. Upon activation, the cytokines bind to the cell surface type 2 TGFβ receptor (TβRII), which further phosphorylates the type 1 TGFβ receptor (TβRI, also known as activin receptor-like kinase, ALK5) and leads to the activation of downstream gene transcription via the Smad-dependent, a canonical pathway. The Smad-independent, non-canonical pathways are NF-κB, p38, MAPK (mitogen-activated protein kinase), Erk (extracellular signal-regulated kinase), TAK1 (TGFβ-associated kinase 1), and Akt signaling pathways. Physiologically, the TGFβ signaling pathway governs key cellular processes during development, tissue regeneration, and regulation of immune responses. Pathological dysregulated activation occurs in cancer and tissue fibrosis-related diseases, providing a strong impetus for the pharmacological modulation of TGFβ signaling. However, difficulty in defining the tissue-specificity and context-dependent nature of TGFβ signaling has challenged the development of clinically useful TGFβ inhibitors [25]. Modern adjuvant therapies antagonize pro-tumorigenic TGFβ signaling by targeting TGFβ ligand production, sequestering and neutralizing ligands, and inhibiting receptor kinase activity. However, with TGFβ being a multifunctional cytokine critical for maintaining tissue homeostasis, on-target anti-TGF-β therapies have been associated with severe side effects, including cardiovascular toxicity and the formation of benign tumors [26]. This suggests that alternated pathways targeting the non-canonical TGFβ pathways may provide better efficiency in combinational therapies.

Interleukin-6 (IL-6) is a pleiotropic pro-inflammatory cytokine that caught attention mainly in chronic inflammation and multifactorial auto-immune disorders. However, overexpression of IL-6 has also been observed in breast, colon, non-small-cell lung, pancreatic, prostate, and ovarian cancers [27] and correlates with poor prognosis [28]. IL-6 is another well-characterized pro-tumorigenic cytokine, which can be present in different stages of cancer development, from cell growth, EMT, migration, and invasion, to metastasis [11,29,30]. Endogenous IL-6 was identified as a resistant factor for some chemotherapeutic agents, such as etoposide and cis-diamminedichloroplatinum, in prostate carcinoma cells [17].

There is increasing evidence that natural compounds from marine organisms have anti-inflammatory and anti-tumor effects [2,8,31,32]. In this study, we examined whether SC-1 inhibits TGFβ-mediated IL-6 and IL-8 expressions in cancer cell lines. Our findings suggest that SC-1 treatment inhibits TGFβ-induced IL-6 release via a non-canonical, p38 pathway. Thus, SC-1 may serve as a lead compound targeting TGFβ-mediated inflammatory diseases and cancer.

## 2. Results

### 2.1. Effects of Sinulariolide on Cell Viability

**Figure 1:**
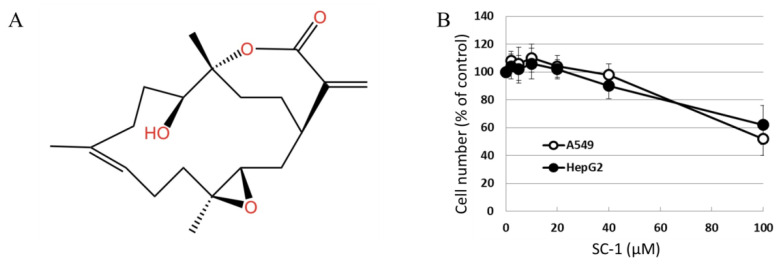
(**A**) Chemical structure of SC-1 (SC-1). (**B**) A549 and HepG2 cells were treated with various dosages of SC-1 (ranging from 1 μM to 40 μM) for duration of 48 h, as indicated. The viability of cells was determined using the CCK8 assay. The data are representative of three independent experiments with mean ± SD (*n* = 3/group).

**Figure 2:**
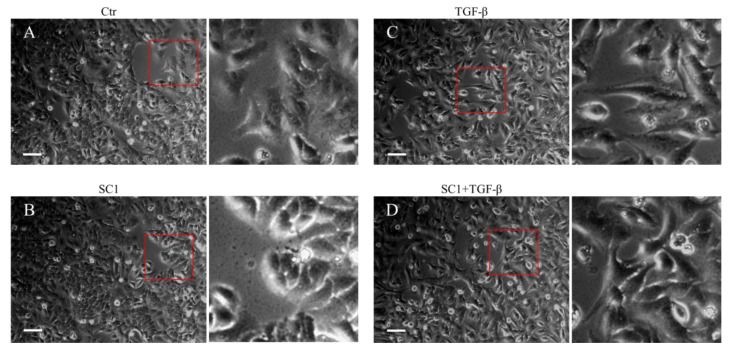
Effects of SC-1 and TGFβ in cell morphology. A549 cells were treated with (**A**) control (DMSO), (**B**) 40 μM SC-1, (**C**) 200 pM TGF-β, and (**D**) 200 pM TGF-β containing 40 μM SC-1 for 24 h. The enlarged red squares are shown in the right-side images for detailed visualization. Bar = 200 μm.

The chemical structure of sinulariolide (SC-1) is illustrated in Figure 1A. SC-1 has been reported to exhibit cytotoxic effects on cancer cells through diverse mechanisms, particularly at higher concentrations [2,4,5]. To ensure that any potential impact of decreased cell viability on cytokine production in SC-1-treated cells is accounted for, we conducted viability assays on A549 and HepG2 cells across a range of SC-1 concentrations. A CCK-8 assay showed that incubating cells with up to 40 μM SC-1 for 48 h did not significantly decrease cell viability (Figure 1B). Therefore, the alteration in protein expression and luciferase activity by SC-1 and TGFβ was not due to a change in cell number and viability. Morphological changes were also used to assess A549 cells following treatment with 40 μM SC-1 and/or 200 pM of TGFβ for 24 h. A549 cells cultured in the control medium displayed a typical cobblestone epithelial morphology. Exposure to SC-1 did not induce any observable morphological changes in A549 cells (Figure 2A,B).

### 2.2. SC-1 Specifically Blocked TGFβ-Induced IL-6 Expression in A549 and HepG2 Cells

**Figure 3:**
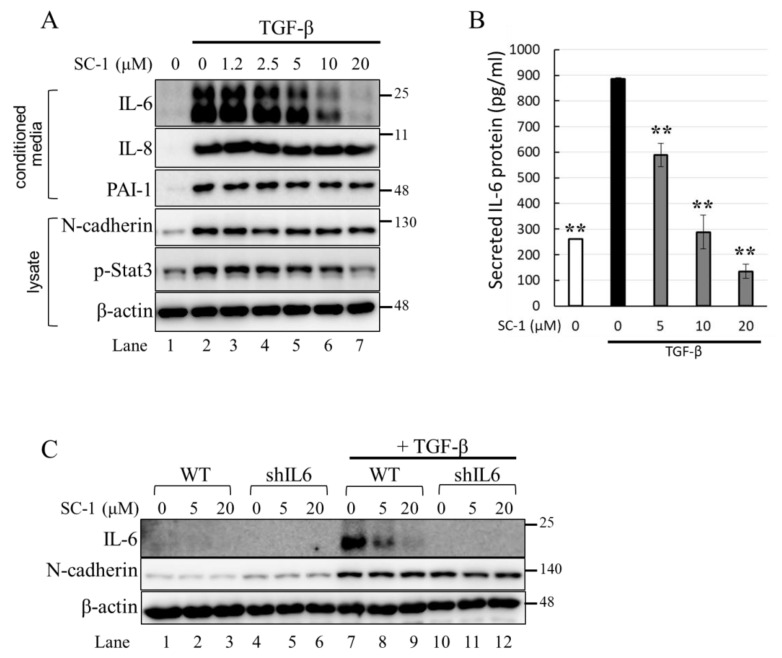
SC-1 specifically inhibited the TGFβ-stimulated IL-6 secretion in A549 cells. (**A**) Levels of IL-6 in the medium were determined by Western blot following incubation of A549 cells in the presence of 1.2, 2.5, 5, 10, and 20 μM SC-1 for 48 h. (**B**) Levels of IL-6 in the medium were detected by ELISA. Each bar represents the mean ± SD of three independent experiments. Statistical significance relative to the TGFβ group is indicated: ** p < 0.001. (**C**) Levels of IL-6 in the medium were detected by Western blot following incubation of A549 cells expressing control or IL-6 silencing shRNA (shIL6) in the presence or absence of SC-1.

We first determined the time profile and concentration required for TGFβ to induce IL-6 production in A549 cells. A549 or HepG2 were used in this study because the cells possess high endogenous TβRII levels and no loss or mutation in other components of the TGFβ signaling pathway [33,34]. Morphologically, a clear transition from cobblestone epithelial to fibroblast-like morphology was observed in cells treated with 200 pM TGFβ for 24 h, indicating the presence of an epithelial–mesenchymal transition (EMT) phenotype (Figure 2C). Importantly, SC-1 did not affect the TGFβ-induced morphological changes (Figure 2D). By using Western blotting, we found that the secretion of the IL-6 protein into the medium was increased by TGFβ stimulation (Figure 3A, lane 2 versus lane 1). To determine whether SC-1 inhibited TGFβ-induced IL-6 secretion, A549 cells were pretreated with SC-1 prior to stimulation with TGFβ. SC-1 pretreatment significantly suppressed TGFβ-induced upregulation of IL-6 in a dose-dependent manner (Figure 3A, lane 3 to 7). To ascertain whether SC-1 broadly inhibits the TGFβ-induced gene expression signature associated with other inflammatory cytokines and the epithelial–mesenchymal transition (EMT) or if it specifically targets IL-6 production, we examined the expression of IL-8, Plasminogen activator inhibitor-1 (PAI-1), and N-cadherin in A549 cells. As shown in Figure 3A, SC-1 did not affect TGFβ-induced EMT protein expression. The IL-6 protein levels in A549 cell culture supernatants were also measured by ELISA after treatment with TGFβ and SC-1. Figure 3B shows that unstimulated A549 cells secreted 260 pg/mL of IL-6, which increased to 886 pg/mL with 200 pM TGFβ treatment, corresponding to a 341% increase (** *p* < 0.001). SC-1 treatment at 5, 10, and 20 μM concentrations led to a decrease in IL-6 protein levels to 589 pg/mL (33% decrease), 288 pg/mL (67% decrease), and 135 pg/mL (84% decrease), respectively (** *p* < 0.001), suggest that the levels of IL-6 protein were suppressed in a concentration-dependent manner as compared with TGFβ alone. In addition, the observed abolition of intracellular STAT3 phosphorylation further supports the inhibitory effect of SC-1 on interleukin-6 production. To provide further confirmation of the identity of the IL-6 protein, we conducted a gene silencing experiment by utilizing shRNA to specifically target and suppress IL-6 expression. As shown in Figure 3C, A549 cells expressing IL-6 shRNA exhibited a complete absence of IL-6, both in the presence and absence of TGFβ (lane 10 compared with lanes 1 and 7). Despite the wider dynamic range and higher sensitivity of ELISA, Western blotting is considered a more specific and accurate method with lower background noise and few false positive results. Therefore, Western blot was used for most IL-6 assays. The decrease in IL-6 secretion was analyzed to determine if it coincided with inhibited mRNA IL-6 levels.

### 2.3. SC-1 Blocked TGFβ-Stimulated IL-6 mRNA Production

**Figure 4:**
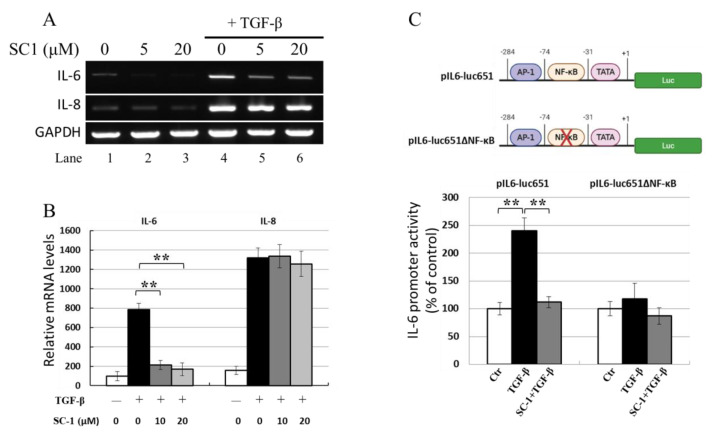
SC-1 inhibited TGFβ-mediated IL-6 promoter activation and mRNA production. A549 cells were pretreated with 0, 5, or 20 μM SC-1 for 4 h, followed by 8 h TGFβ stimulation. Levels of mRNA for IL-6 were assessed by gel-resolving RT-PCR (**A**) and qualitative RT-PCR (qRT-PCR) analysis (**B**). IL-8 mRNA was used as a negative reference, and GAPDH mRNA was used as a control to normalize the total mRNA levels. A reporter assay was performed to analyze IL-6 promoter activity in A549 cells by using a wild-type IL-6 reporter (pIL6-luc651) or an NF-kB-truncated IL-6 reporter (pIL6-luc651∆NF-kB) construct (**C**). Each bar represents the mean ± SD of three independent experiments. Statistical significance relative to the TGFβ group is indicated: ** p < 0.001.

SC-1 reduced TGFβ-stimulated IL-6 protein production in A549 and HepG2 cells; thus, its effect on IL-6 mRNA expression in response to TGFβ was analyzed using gel-resolving RT-PCR (Figure 4A) and qualitative RT-PCR (qRT-PCR) (Figure 4B). Figure 4A demonstrates IL-6 and IL-8 mRNA from cells treated with 100 pM TGFβ for 8 h. The expected size of IL-6 cDNA was 565 bp, and the expected size of the constitutive message for GAPDH serving as an internal control was 225 bp. RT-PCR indicated that TGFβ upregulated IL-6 and IL-8 mRNA, SC-1 specifically inhibits IL-6 mRNA without affecting IL-8 mRNA (Figure 4A, lanes 5 and 6 versus lane 4). The qRT-PCR results in Figure 4B also showed that TGFβ increased IL-6 mRNA levels, which were clearly downregulated by SC-1 treatment (** *p* < 0.001).

### 2.4. SC-1 Inhibited TGF-Mediated the IL-6 Transcription Potentially via an NF-κB-Responsive Element

The effects of SC-1 on the TGFβ-stimulated production of IL-6 were studied using luciferase reporter gene assays. A 651 bp fragment of the human IL-6 promoter was inserted into a luciferase reporter gene vector to generate the pIL6-luc651 construct (Figure 4C). As shown in Figure 4C (lower graph), when transfected into A549 cells, TGFβ (at a concentration of 50 pM) elevated pIL6-luc651 activity to 240 ± 12% of the unstimulated control (as 100%). The addition of 20 μM SC-1 resulted in a reduction in TGFβ-mediated IL-6 promoter activity to 11% (115% versus 240%, ** *p* < 0.001), consistent with the dose-dependent changes observed at the mRNA and protein levels. However, in the cells expressing the reporter construct harboring NF-kB truncation (pIL6-luc651∆NF-kB) [35], IL-6 promoter activity was not changed upon TGFβ treatment.

### 2.5. SC-1 Blocked TGFβ-Induced Cell Migration and Invasion

**Figure 5:**
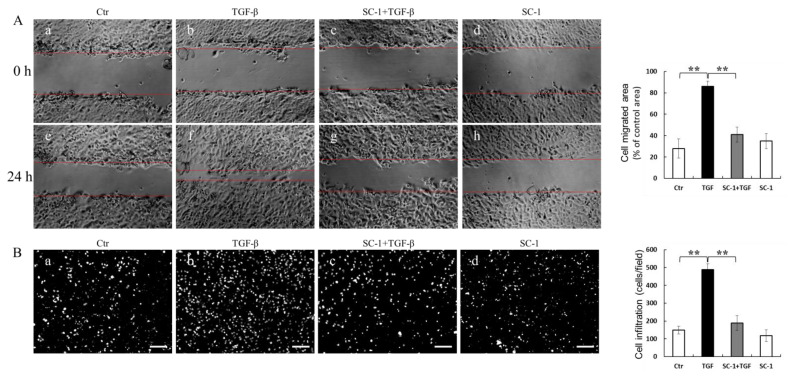
The anti-metastasis-related effects of SC-1 in TGFβ-treated cells. A549 cells were pretreated with SC-1 or equal volume of DMSO for 4 h, wound-healing migration assay (**A**) and transwell invasion assay was performed to analyze the migratory ability of lung cancer cells ((**B**), DAPI staining, *n* = 9). Scale bar represents 200 μm. In migration assay, A549 cells were treated with DMSO (**Ae**) SC-1 (**Af**), TGFβ (**Ag**), or SC-1 plus TGFβ (**Ah**) for 24 h. Wound edges were indicated with red solid lines; results were normalized with 0 h ((**Aa**), (**Ab**), (**Ac**), (**Ad**), respectively) and expressed as percentage of control. In transwell assay, A549 cells were treated with DMSO (**Ba**), TGFβ (**Bb**) SC-1 plus TGFβ (**Bc**), or SC-1 (**Bd**) for 24 h. (**B**) Cells were stained with DAPI and photographed (*n* = 9). Results were represented as number of infiltrated cells based on DAPI signal. Scale bar represents 200 μm. Data are shown as mean ± SD, two-tailed unpaired Student’s *t*-test, ** p < 0.001.

IL-6 has been shown to promote various aspects of tumor progression, such as cell growth, EMT, migration, invasion, and metastasis [30]. In light of this information, we aimed to investigate whether inhibition of IL-6 expression by SC-1 could reduce TGFβ-induced cell migration. The effect of SC-1 on cancer cell migration was assessed using scratch assays and transwell assays in vitro. Briefly, cells were pretreated with a sublethal concentration of SC-1 for 4 h and continued for TGF-β stimulation for 24 h. Compared to cells treated with DMSO (control), TGFβ significantly induced cell migration (Figure 5(Af) versus Figure 5(Ae)). SC-1 suppressed the TGF-β-induced cell migration at a concentration of 20 μM (Figure 5(Ag) versus Figure 5(Af); quantitative result in the right graph). To examine the effect of SC-1 on the invasion of A549 cells, we performed the transwell assays using Matrigel-coated chambers. By performing DAPI staining to observe penetrated cells, we observed a significant reduction in the number of penetrated A549 cells following SC-1 treatment compared to TGF-β-treated cells (Figure 5(Bc) versus Figure 5(Bb), quantitative result in right graph). These results suggest that SC-1 exerts inhibitory effects on the migratory and invasive capacity of cancer cells in vitro.

### 2.6. Using the Cell-Based Assay, p38 MAPK Is Activated by TGFβ and Involved in IL-6 Induction by TGFβ

**Figure 6:**
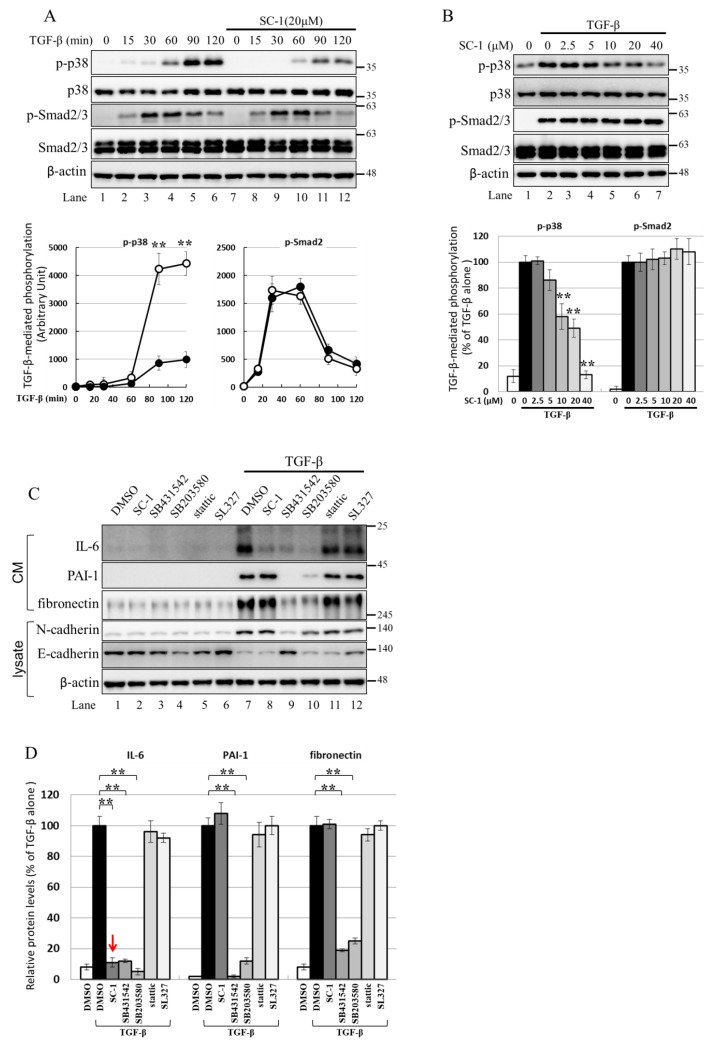
SC-1 specifically inhibited TGFβ-stimulated p38 signaling in A549 cells. (**A**) A549 cells were pretreated with 20 μM SC-1 for 4 h, followed by TGF-β stimulation for different time intervals (15, 30, 60, 90, and 120 min). The quantified results, represented as arbitrary units of signal intensity, are shown in the lower graphs. (**B**) Cells were preincubated with increasing concentrations of SC-1 (0–40 μM) for 4 h, followed by stimulation with TGF-β (100 pM) for 90 min. Protein extraction was performed, and Western blotting was carried out. The protein levels were quantified and presented as a percentage relative to the TGF-β-stimulated control. ** p < 0.001 vs. TGF-β-stimulated control. (**C**) Cells were preincubated with vehicle (DMSO), SC-1 (20 μM), SB431542 (10 μM), SB203580 (10 μM), Stattic (20 μM), and SL327 (20 μM) for 4 h, followed by stimulation with TGF-β (200 pM) for 48 h. The protein levels of IL-6, PAI-1, and fibronectin were quantified in (**D**). The protein level for each gene was normalized to β-actin and expressed as percentage to TGF-β-treated control. Each bar represents the mean ± SD of three independent experiments. Statistical significance relative to TGF-β group is indicated: ** p < 0.001.

The activation of IL-6 by IL-1b or TNF-α is known to be mediated through the p38 MAPK pathway, and p38 MAPK is involved in TGFβ-induced gene expression [36,37,38]. Therefore, we investigated whether SC-1 inhibits the non-canonical TGFβ signaling pathway through p38 MAPK in cancer cells. Figure 6A shows that TGFβ treatment significantly increased the phosphorylation of p38 MAPK and Smad2/3 in A549 cells. The time profile data in Figure 6A demonstrates that TGFβ-induced maximal phosphorylation of p38 MAPK and Smad2/3 occurred at 120 min and 60 min, respectively (lanes 6 and 4). Pretreatment with SC-1 resulted in a marked inhibition of TGFβ-induced p38 phosphorylation without affecting the total expression of p38 (Figure 6A, lane 12 versus lane 6, quantitative result in the lower graph). However, the level of TGFβ-mediated Smad2/3 phosphorylation was not changed by SC-1 treatment (Figure 6A, lane 10 versus lane 4). In addition, SC-1 inhibited TGFβ-induced p38 phosphorylation in a dose-dependent manner (Figure 6A, lane 2 to lane 7) without affecting Smad2/3 phosphorylation (quantitative result in the lower graph). This suggests that p38 MAPK plays a crucial role in IL-6 activation by TGFβ. To elicit the role of p38 in TGFβ-stimulated IL-6 production, TGFβ activation was blocked by treatment with SB431542, which inhibits TGFβ type I receptor (ALK5) kinase activity, by treatment with the p38 inhibitor SB203580, or by SC-1. As shown in Figure 6C,D (a graph of quantitative results), treatment with SB431542, SB203580, or SC-1 resulted in a strong inhibition of TGFβ-induced IL-6 expression at secreted protein levels (Figure 6A, lanes 8, 9, 10 versus lane 7). In contrast, treatment with Stattic, which inhibits STAT3 (Signal transducer and activator of transcription 3), or by MEK Inhibitor SL327 did not inhibit IL-6 production (Figure 6A, lanes 11, 12 versus lane 7).

### 2.7. NF-kB Is Activated by p38 MAPK and Required for TGFβ-Mediated IL-6 Induction

**Figure 7:**
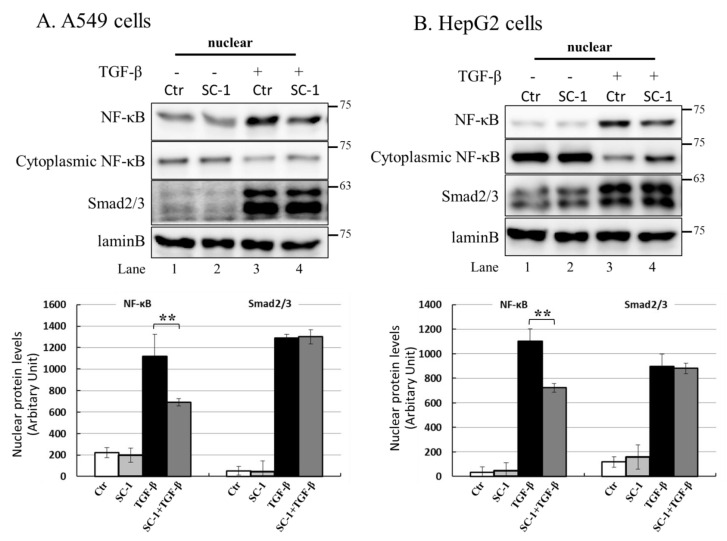

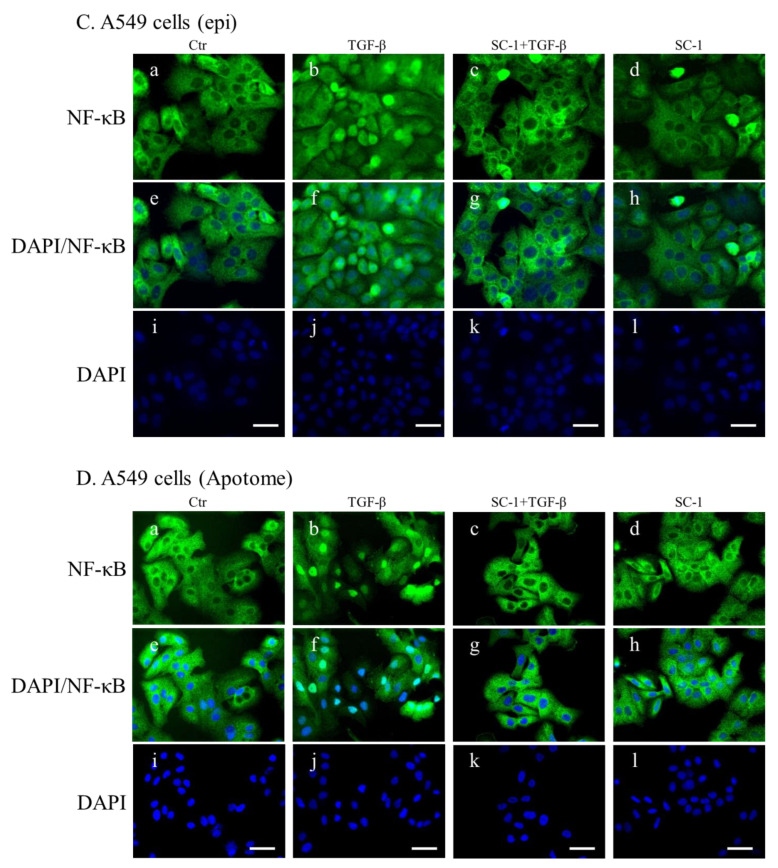
SC-1 inhibited TGF-β-induced NF-kB nuclear translocation. Nuclear translocation of NF-kB proteins was analyzed by separating nuclear fractions after the treatment of A549 (**A**) and HepG2 (**B**) cells with TGF-β or SC-1. Complete fractionation of nuclear proteins and equivalent loading were verified through Western blotting using antibodies against lamin B. Representative images with lower graphs illustrating quantitative analyses of ECL (mean ± SD) from three independent experiments (compared with TGFβ treatment alone); ** *p* < 0.01. In epi-fluorescent micrograph (**C**) and ApoTome microscopy (**D**), images of untreated ((**Ca**) and (**Da**)) and TGF-β-treated ((**Cb**) and (**Db**) are positive controls) A549 cells are shown in comparison with cells treated with 20 μM SC-1 and 100 pM TGF-β ((**Cc**) and (**Dc**)). Immunofluorescence staining was performed as described in the Materials and Methods section. Bar = 100 μM.

NF-kB is a key regulatory factor for IL-6 gene transcription, and its activation by various cytokines occurs through p38 MAPK in many types of human cells [37,39]. As depicted in Figure 4C, the transient transfection of mutant IL-6-luc, which contained a truncation of the NF-kB binding element, exhibited a pronounced inhibition of TGFβ-induced luciferase activity compared to the wild-type IL-6-luc construct. To assess the role of SC-1 in TGFβ-mediated activation of NF-kB, we examined whether SC-1 inhibits TGFβ-stimulated nuclear translocation of NF-kB. Immunoblotting assay for nuclear and cytoplasmic fractions of A549 and HepG2 cells revealed that the nuclear level of NF-kB/p65 is dramatically increased at 20 min after TGFβ treatment (Figure 7A,B). SC-1 treatment ameliorated TGFβ-induced NF-kB/p65 nuclear translocation in A549 and HepG2 cells by 64% and 51%, respectively (Figure 7A,B, lanes 4 versus lanes 3, lower graph). Nuclear translocation of NF-kB was assessed through immunofluorescence microscopy in A549 cells. We observed TGFβ-induced nuclear translocation of NF-kB (Figure 7(Cb) versus (Ca)), which was completely inhibited by pretreatment with SC-1 (Figure 7(Cc) versus (Cb)). To ensure precise detection of the nuclear translocation hindered by SC-1, we employed the ApoTome Structured Illumination system, which effectively removes out-of-focus light. This advanced imaging technique significantly enhances clarity and resolution, facilitating accurate visualization of nuclear translocation events. Consistently, TGFβ-induced nuclear translocation of NF-kB was evident in control cells (Figure 7(Db) versus (Da)), whereas it was abolished by pretreatment with SC-1 (Figure 7(Dc) versus (Db)). Collectively, these findings indicate that TGFβ activates NF-kB through p38 MAPK in lung cancer cells, and SC-1 inhibits TGFβ-mediated IL-6 secretion via the p38–NF-kB signaling cascade.

## 3. Discussion

In this research, we found that the anti-inflammatory and anti-cancer marine natural compound SC-1 blocks TGFβ-induced IL-6 expression. Furthermore, this anti-inflammatory effect of SC-1 is mediated via inhibition of p38 MAPK phosphorylation and then NF-κB activation leading to the mRNA expression associated with pro-inflammatory cytokines, IL-6, in human cancer cells. In this study, we used cell models including non-small-cell lung cancer and hepatocellular carcinoma cell lines because patients with advanced lung cancer and hepatocellular carcinoma express abundant levels of TGFβ ligands and IL-6 in serum, and the TGFβ–IL-6 axis correlates to the tumor development, invasion, and chemoresistance [40,41,42,43,44,45].

Natural compounds have successfully exhibited anti-inflammatory and anti-cancer activities. Numerous compounds reported in several studies are termed anti-IL-6 on the basis of their ability to decrease the IL-6 levels in different cell lines. However, the precise mechanisms responsible for this reduction in IL-6 levels remain unknown. Many chemical substances isolated from plants have been explored to inhibit IL-6 production. Atractylenolide III, the major bioactive component of *Atractylodes lancea*, has been reported to reduce IL-6 secretion by inhibiting the activation of p38 MAPK, C-Jun-N-terminal protein kinase, and NF-κB [46,47]. Xanthoxylin, a plant component in many plants, has been reported for anti-inflammatory activity, and synthetic xanthoxylin derivatives can significantly inhibit IL-6 expression, suggesting that the anti-inflammatory activity of xanthoxylin-rich plants may be a function of xanthoxylin itself [48]. Marine-derived steroids from the Korean sponge *Monanchora* sp have been reported to show potent inhibitory effects on IL-6 production in LPS-stimulated macrophage cells [49]. In addition to natural compounds, a series of imidazoline-based synthetic scaffolds exhibit potent inhibitory effects on NF-κB-mediated gene transcription and suppress TNF-α and IL-6 expression in cultured cell systems [50]. SC-1 in this study is purified from the cultured soft coral *Sinularia flexibilis* and exhibits potent inhibition in TGFβ-induced IL-6 by inhibiting the phosphorylation of p38 and reducing the expression levels of NF-кB. The structure of SC-1 is a cembranolide analog, the chemical skeleton of which differs from that of steroids and has no structure-activity correlation with aforementioned compounds, which may further highlight the potential of SC-1 as a useful therapeutic agent for inflammatory diseases and cancer. The inhibition of TGFβ-mediated cell migration and invasion by SC-1, while not impacting the expression of EMT proteins, is an intriguing observation in this study. Although the exact underlying mechanism remains unclear, we did observe a clue suggesting that SC-1 partially inhibited cell invasion, which may explain why complete elimination of EMT characteristics was not observed. Furthermore, it is possible that IL-6-mediated Stat3 signaling (or other IL-signaling) could serve as an alternative pathway for invasion activation without affecting EMT phenotypes. To further explore this hypothesis, additional investigations are warranted to uncover the specific mechanisms underlying the effects of SC-1 on cell migration, invasion, and EMT protein expression. It is important to note that there are limitations in precisely analyzing cell behavior and invasion in this study, as shown in Figure 2 and Figure 5. By utilizing methods such as electrical impedance measurements, specifically the xCelligence system, we may gain insights into SC-1-mediated cell behavior patterning and reveal EMT-independent cell invasion induced by TGFβ.

Tumor cells and surrounding stroma are responsible for the synthesis of IL-6, which induces inflammation via PI3K, Ras/Raf/MAPK, or Src/YAP pathways through JAK [51]. IL-6 binds to IL-6R, and the IL-6/IL-6R complex activates the JAK/STAT, PI3K/Akt, and Ras/ERK pathways. The human IL-6 promoter contains binding sites for several transcription factors, such as NF-κB, STAT3, C/EBP, CREB, and AP-1, which are known to be involved in the induction of IL-6 gene expression by various cytokines [22,39,52]. The previous study elucidated that activation of NF-kB and AP-1 and their essential role for the IL-6 induction in TGFβ-stimulated cancer cells leads to the hypothesis that TGFβ promotes interaction of these transcription factors with their cis-regulatory elements, which leads to IL-6 mRNA synthesis [17]. In the context of this study, although luciferase assays and immunofluorescent staining indicated that SC-1 inhibited TGFβ-mediated NF-kB activation and consecutive IL-6 expression, we cannot rule out the possibility that SC-1 targets other transcriptional machinery and inhibit IL-6 gene expression. In this respect, CBP/p300 AP-1, C/EBP, or CREB are greatly interested in future prospects [23,53].

TGFβ signaling plays a dual role in tumor progression, acting as a tumor suppressor in the early phase and as a pro-metastatic pathway in the late stages. Accumulating evidence suggests that advanced tumors produce excess TGFβ, which promotes tumor growth, dissemination, and colonization of secondary organs. IL-6 has been reported to affect TGFβ-induced growth inhibition and apoptosis, suggesting that increased IL-6 production by tumor-producing TGFβ may counteract the tumor-suppressive effects of TGFβ hence contribute to the oncogenic conversion of TGFβ function in the malignant progression of hepatocellular carcinoma, lung, and prostate cancers [17,40,44]. Further studies will be interesting to define whether SC-1 alleviates the oncogenic transformation of cancer by blocking TGFβ-induced IL-6 expression. Given that IL-6 plays a more significant role in inflammatory diseases than in cancer development, we intend to explore the therapeutic potential of SC-1 in the treatment of various inflammatory conditions. Several disease models, including rheumatoid arthritis (RA), inflammatory bowel disease (IBD), asthma, dermatitis or psoriasis, and sepsis, can be considered for future investigations into the effects of SC-1 in these inflammatory contexts.

TGFβ has been shown to induce IL-6 expression in various types of cells, including lung fibroblasts, osteoblasts, and cancer cells [17,22,23,24]. Reciprocally, exogenous IL-6 increases TGFβ expression and secretion by various cells [24,54]. In activated human pancreatic stellate cells (PSCs), anti-IL-6 and anti-TGFβ neutralizing antibodies attenuate TGFβ and IL-6 secretion [24], respectively, suggesting that an autocrine loop exists between IL-6 and TGFβ. In addition, IL-6’s diverse effects on TGFβ action have been demonstrated in different aspects of its signaling pathways. In human renal epithelial cells, IL-6 enhances TGFβ signaling by altering the intracellular compartmentalization of TGFβ receptors [55]. Therefore, we postulate that the inhibitory effects of SC-1 might provide an intervention point to arrest the vicious circle of the autocrine feed-forward loops between IL-6 and TGFβ during tumorigenesis and to stimulate possible strategies for cancer treatment.

Although the role of TGFβ in cancer is well studied and efforts to develop therapies to inhibit TGFβ signaling have been ongoing for decades, the progress toward this goal has been limited. Given the pleiotropic nature of TGFβ and its role in maintaining physiological homeostasis, complete inhibition of the TGFβ pathway can lead to serious complications in vivo and on-target toxicities in various tissues, including the cardiovascular system [56]. Therefore, an ideal drug would be able to target multiple levels of the TGFβ downstream pathways while preserving residual TGFβ activity necessary for normal physiological function. One such emerging class of drugs is compounds that modulate the renin–angiotensin system (RAS) [57]. Indeed, this approach has shown promising results in clinical trials for the treatment of chronic wounds. Another advantage of RAS modulation is that it has a broader mode of action, as it also targets mechanisms that impair regeneration that may be, but not necessarily directly, related to TGFβ activity. The current study shows that SC-1 inhibits TGFβ-induced IL-6 secretion by suppressing the p38MAPK and NF-κB pathway without affecting TGFβ receptors and Smad signaling. As shown in Figure 3A, SC-1 effectively inhibits the TGFβ-induced secretion of IL-6 without altering the secretion of EMT-related proteins like PAI-1 and N-cadherin. Interestingly, the commonly used P38MAPK inhibitor, SB203580, not only inhibits IL-6 secretion but also partially attenuates the production of PAI-1 and N-cadherin (Figure 6A). This observation suggests that SC-1 may selectively target a specific subtype of P38MAPK, thereby conferring its specificity for IL-6 inhibition. It is important to note that SB203580 has been reported to potentially exhibit off-target effects, inhibiting other kinases such as c-Jun N-terminal kinase (JNK) and protein kinase A (PKA). To gain further insights into the underlying mechanisms, additional gene-silencing approaches are required to validate and ensure the accuracy of these findings. Several cytokines other than IL-6 are known to be related to the excessive inflammatory responses upon TGFβ stimulation. The limitations of our study are that no cytokines other than IL-6 were evaluated. The specificity of SC-1 preserves the basal TGFβ activity for normal physiological function and prevents IL-6-mediated restoration of cellular sensitivity to TGFβ-mediated growth arrest and apoptosis. The majority of previous studies also tested the therapeutic efficacy of SC-1 by using cell-based and animal models [2,3,4,5,6,7,8,9,31,32,58]. However, it is difficult to deduce from these studies whether the disruption of this pathway affects the initiation and/or maintenance of cancer development. Appropriate mouse models that mimic the advanced stages of human cancers are needed to test the therapeutic efficacy of inhibitors in more clinically relevant settings.

The current understanding of cancer pathogenesis suggests that abnormal expression levels of pro-inflammatory cytokines play an essential role in disease malignancy. Thus, there are significant unmet medical needs in cancer therapy, and small-molecule inhibitors of pro-inflammatory mediators have great potential to address these needs. Although the molecular mechanism of SC-1 action needs to be further clarified, our data strongly suggest that SC-1 inhibits IL-6 production and will provide additional benefit in the management of the disease, which prevents tumor cells resistant to the TGFβ’s tumor suppression activity in cancers.

## 4. Materials and Methods

### 4.1. Reagents and Antibodies

SC-1 was purified and provided by Dr. Jui-Hsin Su from the National Museum of Marine Biology and Aquarium (Pingtung, Taiwan) [32]. SC-1 was dissolved in DMSO as aliquoted stock (10 mM) and then stored at −80 °C. The final concentrations of DMSO in all experiments were lower than 0.1%, and thus, DMSO had no effect on TGFβ signaling [59]. Primary monoclonal antibodies against Samd2/3 (#8685), phospho-Smad2/3 (#8828), p38 (#9212s), phospho-p38 (#4511), NF-κB p65 (#8242), phospho-stat3 (#9145), and PAI-1 (#11907) were purchased from Cell Signaling Technology (Beverly, MA, USA). Primary antibodies against β-actin (sc-47778), N-cadherin (sc-59987), Lamin B (sc-6216), E-cadherin (sc-8426), and fibronectin (sc-18825) were purchased from Santa Cruz Biotechnology. (Dallas, TX, USA). Primary antibodies against IL-6 (AF-206-NA) and IL-8 (MAB208-100) were purchased from R&D Systems (Minneapolis, MN, USA). Secondary antibodies horseradish peroxidase-conjugated goat anti-mouse IgG (#AP124P) and goat anti-rabbit IgG (#AP132P) were obtained from Millipore (Billerica, MA, USA). Alexa Fluor 488 donkey anti-rabbit secondary antibody (A212064) and cell culture materials were obtained from Invitrogen (Carlsbad, CA, USA). Recombinant human TGFβ was purchased from R&D Systems (Minneapolis, MN, USA). The human IL-6 luciferase promoter constructs were provided by Dr. Chih-Hsin Tang (School of Medicine, China Medical University, Taichung, Taiwan). 6-diamidino-2-phenylindole (DAPI) and other chemicals or inhibitors were purchased from Sigma-Aldrich (St. Louis, MO, USA).

### 4.2. Cell Culture

Non-small-cell lung cancer cell line A549 (CCL-185) and hepatocellular carcinoma cell line HepG2 (HB-8065) were purchased from ATCC and cultured according to the suppliers’ instructions. All cells were maintained in Dulbecco’s Modified Eagle’s Medium (DMEM) medium with 10% fetal bovine serum (FBS). Cells were tested and confirmed to be free of mycoplasma, and the cells were subcultured no more than 25 times after thawing.

### 4.3. Preparation of Secretory Proteins and Nuclear Extracts

Conditioned medium (CM) was obtained from 48-h serum-starved cells. They were then centrifuged at 300 g for 10 min (4 °C) and kept at −80 °C until use. The proteins present in the CM were concentrated by the trichloroacetic acid (TCA) precipitation methods, as described in a previous report [60]. The protein recovery yield for each culture condition was assessed via a Bicinchoninic Acid (BCA) Protein Assay kit from Thermo Fisher Scientific (Waltham, MA, USA). IL-6 present in the CM was measured by using Western blot and the Quantikine IL-6 ELISA Kit (R&D Systems, Carlsbad, CA, USA). Nuclear proteins were extracted from A549 cells using Nuclear and Cytoplasmic Extraction reagents (Pierce; Thermo Fisher Scientific, Waltham, MA, USA) according to the manufacturer’s protocol. The samples were stored at −80 °C for the analysis of NF-κB and Smad2/3 proteins. The protein levels of NF-κB and Smad2/3 were measured in the nuclear protein extracts (15 µg) using Western blot.

### 4.4. Western Blotting

For Western blotting, total proteins from A549 cells were first lysed using a RIPA buffer containing 25 μg/mL phenylmethylsulfonyl fluoride. The cell lysate was then incubated on ice for 30 min before being centrifuged at 14,000 rpm for 10 min at 4 °C. Protein concentrations were quantified with a BCA Protein Assay kit. Equal amounts of protein (25 μg) were separated by 10% SDS-PAGE and transferred onto polyvinylidene difluoride membranes. After blocking with 5% non-fat milk for 2 h, membranes were incubated with primary antibodies at 4 °C for 24 h. Subsequently, membranes were incubated with horseradish peroxidase-conjugated secondary antibodies at room temperature for 1 h. The resulting immunoreactive bands were visualized using an ECL detection system (Amersham Biosciences, Buckinghamshire, UK), and images were acquired using an ImageQuant™ LAS 4000 (GE Healthcare, Chicago, IL, USA). Quantification of images was performed by ImageJ software (version 1.43; National Institutes of Health, Bethesda, MD, USA).

### 4.5. Immunofluorescent Staining

A549 cells were grown on 25 mm cover glasses and treated with 100 pM TGFβ in the presence or absence of SC-1 at varying concentrations. After the treatment, the cells were fixed in 4% PFA for 15 min. Next, the cells were washed three times with cold PBS, permeabilized with 0.5% Triton X-100 for 10 min, and washed three times again with cold PBS. The cells were then blocked in immunofluorescent (IF) blocking buffer for 3 h, followed by incubation with NF-κB antibody (1:800) in IF blocking buffer for 2 h at room temperature. The secondary antibody was then diluted in IF blocking buffer and incubated for 2 h at room temp. The secondary antibody was removed and replaced with 1 × PBS and counter-stained with 1 μg/mL DAPI for nuclear staining for 10 min RT with gentle rocking. The cover glasses were then washed 4 times for 10 min with 1X PBS, and the cells were imaged using the Zeiss Axio Observer Z1 equipped with an ApoTome. In brief, 9 fields of view from each well were imaged using 2 channels. The nuclei were identified and counted in channel 1. The relative fluorescent intensity and area were quantified and used to calculate the expression levels of NF-κB per cell in channel 2. The data are represented as the mean values obtained from 3 individual replicates.

### 4.6. Cell Viability

Cytotoxicity of SC-1 was tested with a Cell Counting Kit-8 (CCK-8; Dojindo, Tokyo, Japan) according to the manufacturer’s protocol. A549 cells were grown on 96-well plates for 24 h. In addition to an untreated control group, SC-1 (2, 5, 10, 40, or 100 μM) was added to cells in the FBS-free DMEM. After 48 h, the cells were incubated at 37 °C for 1 h with 10 μL of CCK-8 working solution. Optical density (OD) values were detected with SpectraMax ID3 (Molecular Devices, LLC. San Jose, CA, USA) at 450 nm.

### 4.7. Enzyme-Linked Immunosorbent Assay (ELISA)

Protein levels of IL-6 in culture supernatants were detected by ELISA. A549 cells were grown in 12-well plates. After preincubation with SC-1 for 4 h and stimulation with TGFβ for 48 h, culture supernatants were recovered, and the protein levels were measured by Quantikine IL-6 ELISA Kit (Boster Bio, Wuhan, China) according to the manufacturer’s protocols. Data were expressed in terms of mean ± SD, and statistical significances were analyzed by *t*-tests.

### 4.8. Cell Migration Assays

A549 cells (2 × 10^4^) were grown in each well of a 24-well cluster plate and incubated at 37 °C with 5% CO_2_ overnight. A549 cells were pretreated with 20 μM SC-1 for 4 h. The cell layer was then scraped using a 200 μM pipette tip and washed to remove debris and followed by a fresh medium containing 0.5% serum. Cells were then incubated with 200 pM of TGFβ for 24 h. Cell migration was determined by measuring wound closure. Representative photographs were taken at ×200 magnification using a NIKON TE2000-U inverted microscope (Nikon, Tokyo, Japan).

### 4.9. Transwell Migration Assay

A549 or HepG2 cells were subjected to treatment with 20 μM SC-1 in the presence or absence of TGFβ for 24 h. A total of 2 × 10^4^ cells were suspended in serum-free medium and seeded into the inserts of transwell chambers (Greiner, Kremsmünster, Austria). The lower chambers were filled with medium containing 10% FBS, and the cells were incubated at 37 °C. After treatment, the migrated cells were fixed using methanol and stained with DAPI. Images of the migrated cells were captured at ×200 magnification using a Zeiss Axio Observer inverted microscope (Zeiss, Oberkochen, Germany). The DAPI intensity of the migrated cells was analyzed using a Molecular Devices Spectramax fluorometer (Molecular Devices, San Jose, CA, USA).

### 4.10. Cell Transfection and Luciferase Assays

A549 cells grown on 24-well plates (2 × 10^4^ cells/well) were serum-deprived for 24 h and subjected to transfection using Lipofectamine 3000 (Thermo Fisher Scientific) for 2 h [35]. Cells were then overlaid with low serum medium with or without SC-1 at the indicated concentrations. After 4 h of preincubation, cells were continued to stimulate with 50 pM TGFβ for 24 h. Cells were harvested by active lysis, and equal amounts of lysates were analyzed for firefly luciferase expression (Dual Luciferase Assay, Promega) according to the manufacturer’s instructions. In brief, 100-μL aliquots of cell lysates were mixed with 50-μL of luciferase reagent buffer, and luminescence of the samples was integrated over a time period of 10 s in a SpectraMax ID3 (Molecular Devices, LLC. San Jose, CA USA). Expressing plasmids encoding Renilla luciferase driven by the thymidine kinase promoter (0.4 mg/well) was used as an internal control.

### 4.11. Reverse Transcription Quantitative Polymerase Chain Reaction (RT-qPCR) and Quantitative Polymerase Chain Reaction (qPCR)

RNA isolation was performed using TRIzol (Thermo Fisher Scientific). A total of 2 µg RNA was reverse transcribed using M-MLV reverse transcriptase (Promega Corporation, Madison, WI, USA) to cDNA in a 20 µL final reaction volume containing M-MLV 5X Reaction Buffer, 10 mM dNTP, 500 µg/mL oligo dT15 primers, and nuclease-free water, which were incubated at 42 °C for 15 min. A total of 100 pg of cDNA was used to initiate qPCR, which was performed using the SYBR-Green PCR Master Mix (Thermo Fisher Scientific) in the ABI 7500 Fast Real-Time system (Thermo Fisher Scientific). The primer pairs used were as follows: inducible IL-6, forward 5′-ACATCGACCCGTCCACAGTAT-3′ and reverse 5′-CAGAGGGGTAGGCTTGTCTC-3′; GAPDH, forward 5′-CGTGTTCCTACCCCCAATGT-3′ and reverse 5′-TGTCATCATACTTGGCAGGTTTCT-3′. GAPDH was used to normalize transcription and calculate the fold induction relative to controls without TGFβ or SC-1 treatment.

### 4.12. Statistical Analysis

One-way analysis of variance (ANOVA) was used for the comparison of more than two mean values. Results represent at least two to three independent experiments and are shown as averages ±SD. Results with a *p*-value of less than 0.001 were considered statistically significant.

## Data Availability

Not applicable.
